# Drug Content Effects on the Dispersion Performance of Adhesive Mixtures for Inhalation

**DOI:** 10.1371/journal.pone.0071339

**Published:** 2013-08-14

**Authors:** Floris Grasmeijer, Paul Hagedoorn, Henderik W. Frijlink, Anne H. de Boer

**Affiliations:** Department of Pharmaceutical Technology and Biopharmacy, University of Groningen, Groningen, The Netherlands; Massey University, New Zealand

## Abstract

The drug content in adhesive mixtures for inhalation is known to influence their dispersion performance, but the direction and magnitude of this influence depends on other variables. In the past decades several mechanisms have been postulated to explain this finding and a number of possible interacting variables have been identified. Still, the role of drug content in the formulation of adhesive mixtures for inhalation, which includes its significance as an interacting variable to other parameters, is poorly understood. Therefore, the results from a series of drug detachment experiments are presented in which the effect of drug content and its dependence on flow rate, the mixing time and the type of drug is studied. Furthermore, it is investigated whether the effect depends on the range within which the drug content is changed. Quantitative and qualitative multiple order interactions are observed between these variables, which may be explained by a shifting balance between three different mechanisms. The results therefore demonstrate that accounting for (multiple order) interactions between variables has to be part of quality by design activities and the rational design of future experiments.

## Introduction

The therapeutic doses of the drugs used in the local treatment of asthma and COPD differ substantially. For example, the long-acting beta agonist formoterol and the anticholinergic tiotropiumbromide are usually administered in doses from 6 to 18 µg, whereas for the corticosteroid fluticasone doses from a single inhalation up to 500 µg are given. The large variation in doses is reflected in the drug contents that are present in commercially available inhalation powder formulations. For adhesive mixtures drug contents typically range from 0.1 to 4% (Tables 1 and 2).

**Table 1:** Drug contents in commercially available carrier based dry powder formulations containing a single drug.

Preparation	Drug	Carrier/dose	Drug/dose	Drug content
Eklira Genuair 400	ACL	12600 µg	400 µg	**3.08%**
Beclometason 100 Cyclocaps	BDP	25000 µg	100 µg	**0.40%**
Beclometason 200 Cyclocaps	BDP	25000 µg	200 µg	**0.79%**
Beclometason 400 Cyclocaps	BDP	25000 µg	400 µg	**1.57%**
Budesonide Easyhaler 200	BUD	7903 µg	200 µg	**2.47%**
Budesonide Easyhaler 400	BUD	14507 µg	400 µg	**2.68%**
Budesonide Novolizer 200	BUD	10700 µg	200 µg	**1.83%**
Budesonide Novolizer 400	BUD	10500 µg	400 µg	**3.67%**
Flixotide Diskus 100	FP	12500 µg	100 µg	**0.79%**
Flixotide Diskus 250	FP	12500 µg	250 µg	**1.96%**
Flixotide Diskus 500	FP	12500 µg	500 µg	**3.85%**
Formoterol Novolizer 6	FFD	5744 µg	6 µg	**0.10%**
Formoterol Novolizer 12	FFD	11488 µg	12 µg	**0.10%**
Formoterol Easyhaler 12	FFD	8000 µg	12 µg	**0.15%**
Seebri Breezhaler	GLY	23600 µg	63 µg	**0.27%**
Onbrez Breezhaler 150	IM	24800 µg	194 µg	**0.78%**
Onbrez Breezhaler 300	IM	25000 µg	388 µg	**1.53%**
Salbutamol Cyclocaps 200	SS	25000 µg	200 µg	**0.79%**
Salbutamol Cyclocaps 400	SS	25000 µg	400 µg	**1.57%**
Salbutamol Novolizer 100	SS	11420 µg	120 µg	**1.04%**
Ventolin Diskus 200	SS	40000 µg	200 µg	**0.50%**
Serevent Diskus	SX	12500 µg	50 µg	**0.40%**
Spiriva Handihaler 18 µg	TBM	5500 µg	22.5 µg	**0.41%**

ACL = aclidinium bromide; BDP = beclometasone dipropionate; BUD = budesonide; FP = fluticasone propionate; FFD = formoterol fumarate dihydrate; GLY = glycopyrronium bromide; IM = indacatarol maleate; SS = salbutamol sulphate; SX = salmeterol xinafoate; TBM = tiotropium bromide.

**Table 2:** Drug contents in commercially available carrier based combination preparations.

Preparation	Drug 1	Drug 2	Carrier/dose	Drug 1/dose	Drug 2/dose	Drug 1	Drug 2	Total content
Foster NEXThaler	BDP	FFD	9894 µg	100 µg	6 µg	1.00%	0.06%	**1.06%**
Seretide Diskus 50/100	FP	SX	12500 µg	100 µg	50 µg	0.79%	0.40%	**1.19%**
Seretide Diskus 50/250	FP	SX	12500 µg	250 µg	50 µg	1.95%	0.39%	**2.34%**
Seretide Diskus 50/500	FP	SX	12500 µg	500 µg	50 µg	3.83%	0.38%	**4.21%**

BDP = beclometasone dipropionate; FP = fluticasone propionate; FFD = formoterol fumarate dihydrate; SX = salmeterol xinafoate.

In contrast to the wide range of drug contents in marketed inhalation products, it is almost common practice to use a fixed drug to carrier ratio of 1∶67.5 (1.46%) in studies on the relationship between certain variables and carrier based formulation performance. This ratio is most likely adopted from earlier carrier based formulations for the Rotahaler®, Diskhaler® and Cyclohaler [1]. Therefore, the choice for this particular drug to carrier ratio seems to be based on historical reasons, rather than it having a sound scientific foundation.

Such a scientific foundation may be found in several studies which have already shown that drug content affects blend homogeneity and drug detachment from the carrier. Adhesive mixtures for inhalation can be considered ‘total mixes’, in which a dynamic process of ordering and randomisation determines the outcome of the blending process [2]. With increasing drug content the equilibrium is displaced towards randomisation [2]–[4], which results in less homogeneous mixtures that are more prone to segregation during handling as well [5]. On the upside, this adverse effect may come along with better drug detachment from the carrier particles when separation forces are generated, as was reported for a model interactive system containing smooth spherical carrier particles [6]. For inhalation formulations that contain lactose carriers the reported effects of drug content on dispersion performance are not consistent, however. Beneficial effects of an increasing drug content from 0.25 to 2.8% on the fine particle fraction (FPF) from two commercially available inhaler devices were reported by Steckel et al [7], whereas other evaluation studies described unfavourable effects as well [8], [9].

In explaining the effect of the drug content on the dispersion performance of adhesive mixtures for inhalation several mechanisms have been postulated. Firstly, so-called active sites on the lactose carrier surface may become saturated with increasing drug content [10], [11]. This results in a decreasing mass percent of drug adhering strongly to the carrier surface, which improves drug particle detachment. Secondly, layer formation or agglomeration of drug particles with increasing drug content can cause detachment of large agglomerates rather than single drug particles if failure of adhesive bonds between the inner drug layer and the carrier surface occurs during inhalation [6], [12]–[14]. This too may improve drug detachment, because it increases the magnitude of the lift and inertial separation forces more so than that of the drug-carrier interaction forces. Thirdly, a higher drug content increases the effectiveness of inertial and frictional mixing forces as drug particles fill up clefts and depressions in the carrier surface [14], [15]. By being transferred through the powder bed, this enables such mixing forces to act effectively as press-on forces on drug particles that would have found shelter from them in carrier surface irregularities at a much lower drug content. It causes the particle interaction forces to increase by reducing their separation distance and increasing their contact surface area, and therefore, it causes drug detachment to be negatively affected [16]. And lastly, it was hypothesised that with increasing drug content detached particles may be more likely to collide with neighbouring drug particles to cause enhanced drug detachment; a mechanism which is referred to as the ‘collision effect’ [6]. Collision effects may marginally contribute to improved drug detachment with increasing drug content, but particularly for irregular lactose surfaces their relevance is questionable. Therefore, they are not further discussed in this paper.

Since not all of the aforementioned mechanisms result in improved drug detachment, the net effect of a change in drug content depends on which mechanism is dominant under the given circumstances. Therefore, rather the balance of a number of different mechanisms determines the effect of drug content than either of these mechanisms alone, and any variable by which this balance is affected will be an interacting variable. Some variables that are suspected to interact with the effect of drug content are the range within which the drug content is changed [8], [9]; the dispersion principle used and its efficacy (e.g. flow rate) [14], [15]; the mixing conditions applied; and the characteristics of the used carrier product [15]. In addition, agglomerate formation may be drug dependent, and therefore, so may be the effect of drug content.

A better understanding of the influence of drug content on dispersion performance may lead to a more rational approach in future studies regarding the choice for this variable. Furthermore, it would help to understand the functionality and performance of carrier products and marketed formulations and to explain the results from powder dispersion performance tests that are presented in literature. The objective of this study is therefore to deepen the understanding of the effect of drug content on drug particle detachment from lactose carriers during inhalation. To this end, a series of drug detachment experiments is performed with formulations containing a range of drug contents. A special focus is put on the role of flow rate, the type of drug, the range within which the drug content is changed and mixing time as interacting variables. Scanning electron microscopy (SEM) and laser diffraction analysis are used in an attempt to qualify and quantify the occurrence of the different mechanisms behind the effect of drug content.

## Materials and Methods

### Starting Materials

Alpha lactose monohydrate (Pharmatose 80 M) was kindly donated by DFE Pharma (Goch, Germany). The micronised drugs used for this study are salbutamol sulphate (supplied by DFE Pharma), salmeterol xinafoate and fluticasone propionate (granted by Novartis, Germany) and budesonide (purchased from Fagron, The Netherlands). All drugs were passed through a 90 µm sieve to break up larger agglomerates prior to mixing with the carrier.

### Carrier Classification

A lactose carrier with a size fraction of 250–315 µm was obtained from Pharmatose 80 M by 20 minutes of vibratory sieving at an amplitude of 1.5 mm (Retsch AS 200 control, Germany) followed by 15 minutes of air jet sieving (Alpine AS200, Augsburg, Germany). Such a coarse size fraction of the crystalline carrier material stresses the effect of press-on forces during the mixing process. It is therefore expected to aid in identifying and determining the relative significance of this mechanism behind the effect of drug content on drug detachment. Sieving was performed under uncontrolled environmental conditions (with relative humidity values ranging from 25–65%). After sieving, the lactose was left to rest for at least 2 days before further processing to allow electrostatic charges to dissipate.

### Blend Preparation

Drug-carrier blends were prepared in batches of 25 g at ambient conditions. To prevent electrostatic charge effects as much as possible, a stainless steel mixing vessel with a volume of 160 cm^3^ was used (the filling degree was approximately 20%). During filling of the vessel the drug was ‘sandwiched’ in between two equal parts of the lactose carrier after which the powder was gently pre-blended with a spatula for approximately 20 orbits. Blends were then further mixed in a Turbula blender (WA Bachhofen, Basel, Switzerland) at 90 rpm for a total of 2, 10 or 60 minutes. Every two minutes any powder adhering to the mixing vessel wall was scraped loose and returned into the blend. For salmeterol and fluticasone, blends containing 0.1, 0.2, 0.4, 1, 2 and 4% of drug by weight were prepared, whereas for salbutamol and budesonide only drug contents of 0.4% and 4% were used. These drug contents are representative for the typical range found in commercial preparations (Tables 1 and 2). However, it is important to realise that the nature of particle interactions in adhesive mixtures for inhalation is ultimately determined not so much by the drug content as by the drug particle density on the carrier surface. This can be expressed as the drug mass per unit carrier surface area (i.e. carrier surface payload) or the degree of carrier coverage relative to a single monolayer. This means that a comparison between mixtures based on drug content is only valid if they contain a lactose carrier with the same specific surface area (and a comparable surface roughness and surface ‘activity’). As will become clear from the calculated drug contents for monolayer carrier coverage presented in Table 3, drug contents in this study are chosen such that carrier coverage values range from 6% (for the 0.1% fluticasone mixture) to 328% (for the 4% budesonide mixture), representing sub-monolayer to multilayer carrier coverage. It should be noted that these values for the carrier coverage are based on the assumption of a uniform drug distribution over the carrier surface, which is unlikely to represent the situation in practice. Therefore, they should merely be regarded as a rough indication for the average drug particle density on the carrier surface.

**Table 3:** PSDs (n = 2) and densities (n = 4) of the drugs.

Drug	X_10_	X_50_	X_90_	V% ≤5 µm	ρ (g/cm^3^ (SD))	Monolayer (%)*	m_particle_ (10^−12^ g)**
Salmeterol	0.66	1.41	3.04	99.56	1.23 (0.004)	1.24	1.81
Fluticasone	0.72	1.80	3.99	95.66	1.36 (0.007)	1.75	4.15
Salbutamol	0.63	1.24	2.60	100	1.30 (0.012)	1.16	1.30
Budesonide	0.65	1.36	2.90	99.96	1.25 (0.008)	1.22	1.81

* The drug content for theoretical monolayer coverage is calculated as explained previously [15].

** Drug particle mass is calculated based on the X_50_ and density of the drugs.

### Content Uniformity Testing

Content uniformity of the blends was tested by randomly taking 20 samples of 25±1 mg. Samples were analysed as explained in the section ‘sample analysis’. Content uniformity was considered acceptable at relative standard deviations (RSDs) <3%.

### Helium Pycnometry

The density of the drugs was measured by helium pycnometry (model MVP-1, Quantachrome, USA). Sample sizes ranged between 5.75 g and 12.35 g. Results are the mean of 4 measurements.

### Laser Diffraction Analysis

The particle size distributions (PSDs) of the drugs were determined by laser diffraction technique with the HELOS BF diffractometer (100 mm lens, calculations based on the Fraunhofer theory) after dispersion of the powders with a RODOS disperser at 3 bar (Sympatec, Clausthal-Zellerfeld, Germany). Increasing the pressure drop to 5 bar did not have any influence on the PSDs measured, confirming that the primary PSDs were already obtained at the lower pressure.

The PSDs of salmeterol and fluticasone particles (including agglomerates) as present on the carrier surface in mixtures containing 0.1% to 1% of drug were measured by wet laser diffraction. To prepare saturated aqueous drug solutions, suspensions of fluticasone and salmeterol containing 0.03% polysorbate 80 (Tween 80) were prepared by ultrasonication for at least 30 minutes with a Helma Transsonic 700/H ultrasonic bath (Elma Hans Schmidbauer, Singen, Germany) and subsequent continuous stirring. After cooling down to room temperature, 35–40 mL of these suspensions was passed through a 0.2 µm cellulose acetate filter. To the saturated solutions 20 mg (for 1% mixtures) to 200 mg (for 0.1% mixtures) of the blends was added to dissolve the lactose carrier and suspend the drug. For the sample sizes used, optical concentrations ranged between 6 and 14%. It was checked that dissolved lactose did not influence the results. Measurements were performed in the CUVETTE SC-40 module (50 mL cuvette) with the HELOS BF diffractometer with a 100 mm lens and the FREE computation mode (Sympatec, Clausthal-Zellerfeld, Germany). A stirring speed of approximately 500 rpm in the cuvette was used continuously to prevent sedimentation of the suspended drug particles. After 12 minutes all lactose particles were fully dissolved for all samples and, immediately afterwards, the PSD of the suspended drug particles was measured for 10 seconds. The primary PSDs of fluticasone and salmeterol were measured in a similar way after a pre-suspension step comprising sonication of approximately 0.5 mg of the drugs in 2 mL of the saturated solution for 11 minutes using a 70 W, 42 kHz ultrasonic cleaner (Electris UC449UP, France). The amount of pre-suspension that was added to the CUVETTE was titrated to an optical concentration of around 10%. It was checked that the optical concentration and characteristic PSD data of the primary particles thus measured remained constant for at least 12 minutes. Because wet suspension methods as described here are sensitive to bias especially from (de-)agglomeration effects, the reliability of the data from these measurements will be discussed further on.

### Scanning Electron Microscopy

Scanning electron micrographs of the drugs and blends were taken with a JSM-6301F (Jeol, Japan) at an acceleration voltage of 3 kV and probe current 7. Samples were fixed to an aluminium specimen mount by means of double sided adhesive carbon tape. For the pure drugs any excess sample was blown from the tape with pressurised air and excess particles from the blends were gently tapped (instead of blown) from the specimen mount to avoid drug particle detachment from the carrier crystals. The drugs were sputter coated with 10 nm of a gold-palladium alloy, whereas for the mixture samples a coating thickness of 20 nm was found to be necessary to prevent charging effects.

### Drug Detachment Experiments

An air classifier based test inhaler was used for the drug detachment experiments [17]. The design of the classifier used is such that >95% of the carrier particles is retained during the dispersion measurement. The residual amount of drug present on the surface of the retained carrier particles after a dispersion experiment was analysed and normalised to 100% retention (referred to as carrier residue, CR). The percentage of drug detached was calculated as 100-CR. Note that drug detachment in this study refers to the relative amount of drug detached. Therefore, if drug detachment is concluded to be deteriorated with increasing drug content, the absolute amount of drug detached may have increased nonetheless. A dose weight of 25±1 mg was used for the dispersion tests and all results presented are the mean of 5 measurements. All measurements were performed at ambient conditions and with a fixed suction time of 3 seconds at 10 to 80 L/min (corresponding to pressure drops of up to 20.1 kPa). Such high pressure drops are not representative for the pressure drops to be attained by patients across dry powder inhalers [18]. However, the purpose of this study was not to simply conduct functionality experiments under conditions relevant to the practice of dry powder inhalation. Rather, the aim is to obtain a mechanistic insight into the effect of drug content, and it will become clear from the results presented in this paper that this requires detachment to almost complete removal of all drug particles from the carrier surface. Finally it is to be noted that changes in drug detachment that are measured in this way do not always correlate to fine particle fractions, since large drug agglomerates may be detached too. However, because the first step to obtaining finely dispersed particles is drug detachment, understanding the mechanisms behind drug particle liberation from the carrier is highly relevant.

### Sample Analysis

Collected samples of the salmeterol, fluticasone, budesonide and salbutamol blends and carrier residues were spectrophotometrically analysed at wavelengths of 228, 228, 243 and 225 nm, respectively (Unicam UV-500, ThermoSpectronic, Cambridge, UK). Salbutamol was dissolved in demineralised water and all other drugs in ethanol. Samples were allowed to dissolve for at least one hour. Prior to UV-analysis the samples in ethanol were centrifuged for 5 minutes at 3000 rpm to obtain solutions free from suspended lactose (Hettich Rotanta D-7200, Hettich AG, Switzerland). Absorption values were corrected for any absorption caused by the dissolution of lactose when applicable.

### Statistical Analysis

A heteroscedastic 2-tailed Student’s t-test was performed using Microsoft Excel 2010 to test the significance of the difference in drug detachment at 20 and 50 L/min between formulations containing 0.4% and 4% of drug.

## Results

### Content Uniformity

The highest RSD in drug content measured was 2.04%, and therefore all blends prepared were sufficiently homogeneous and considered suitable for the dispersion experiments.

### SEM Imaging

**Figure 1:**
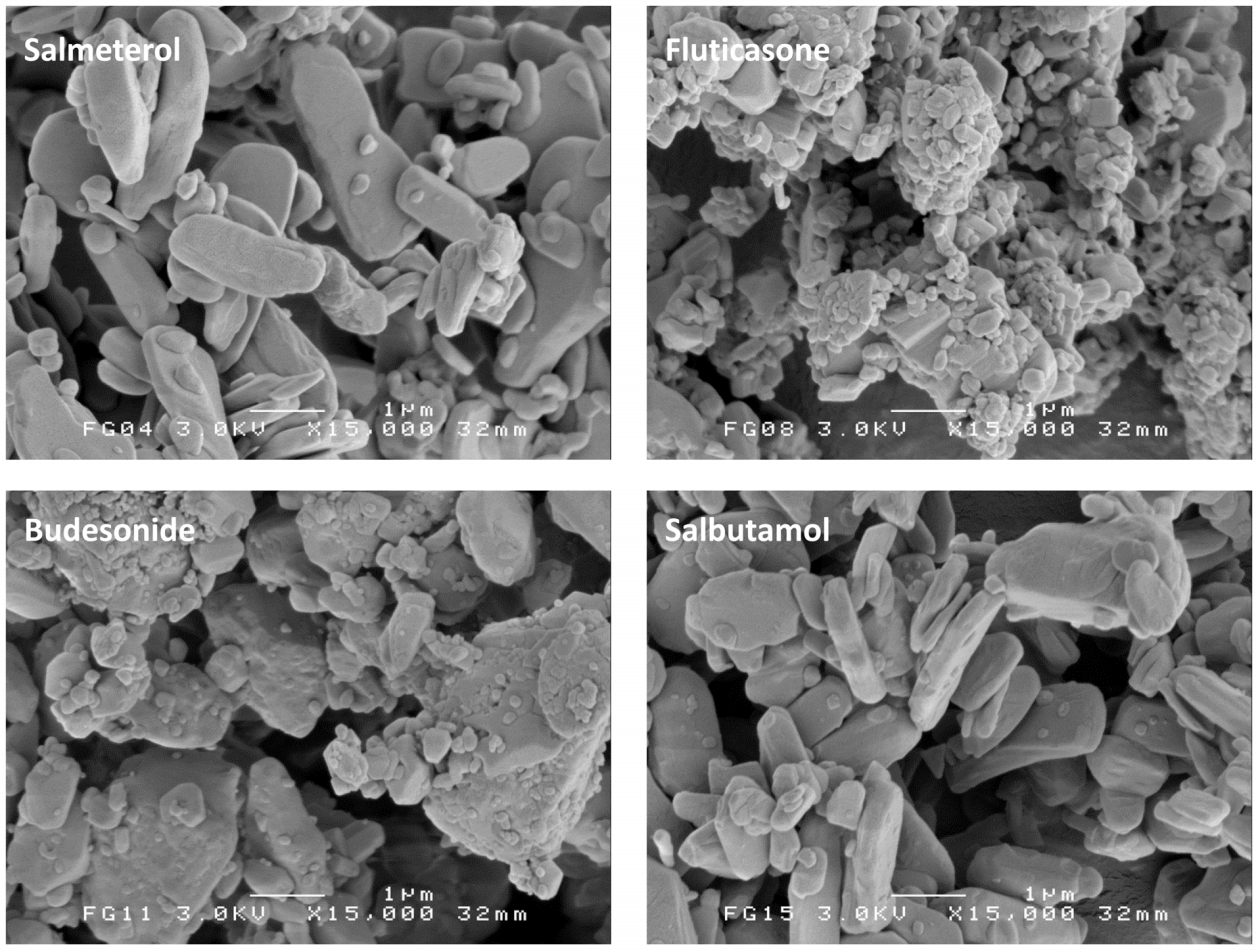
Representative SEM images of the four micronised drugs.

SEM images presented in Figure 1 show that both bronchodilator drugs salmeterol and salbutamol consist of plate like particles, whereas the corticosteroids budesonide and fluticasone are more irregularly shaped. In addition, the corticosteroids seem to contain more submicron particles, although this is not reflected in the X_10_ values obtained from RODOS dispersion (Table 3). This suggests that these fines are firmly attached to the coarser particles or comprise only a negligible volume fraction.

**Figure 2:**
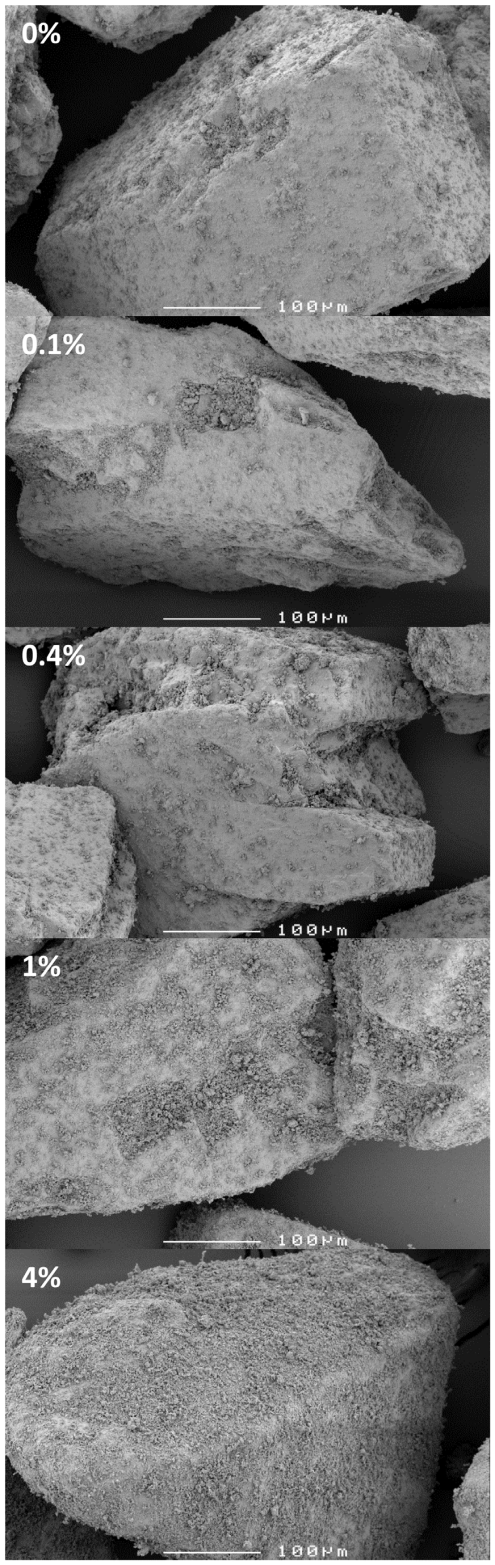
Representative SEM images of the carrier after the mixing process (0%) and of salmeterol blends. The percentage indicates the salmeterol content of the mixture.

The lactose carrier contains a notable amount of adhering lactose fines after being subjected to the same mixing conditions as the blends, despite the preceding classification procedure (Fig. 2, 0%). It is difficult to distinguish between these fines and the drug particles, particularly at a relatively low magnification of 250×(Fig. 2, 0.1–4%). This limits the possibility to accurately monitor the spatial distribution of the drug particles over the carrier surface with SEM. At salmeterol contents of 1% and higher, carrier surface irregularities become filled up and covering of the carrier surface by drug particles becomes more apparent. For the other drugs the observations from SEM were similar.

**Figure 3:**
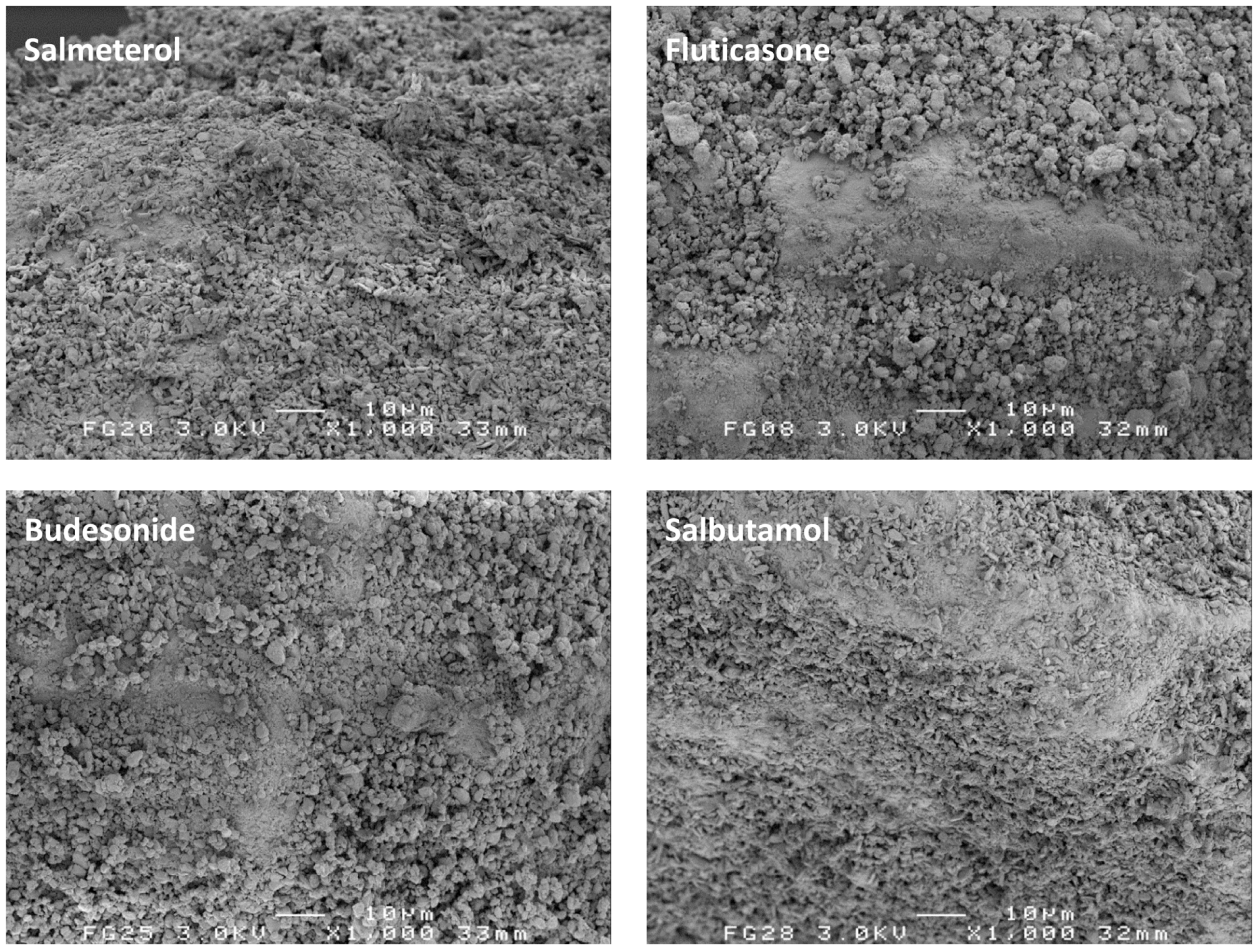
Representative SEM images of the 4% mixtures for the different drugs.

In Figure 3, representative carrier surface close-ups of 4% mixtures are shown at the same magnification. No notable differences in the degree of agglomeration between the different drugs can be observed.

### Pycnometry and Laser Diffraction Analysis

**Figure 4:**
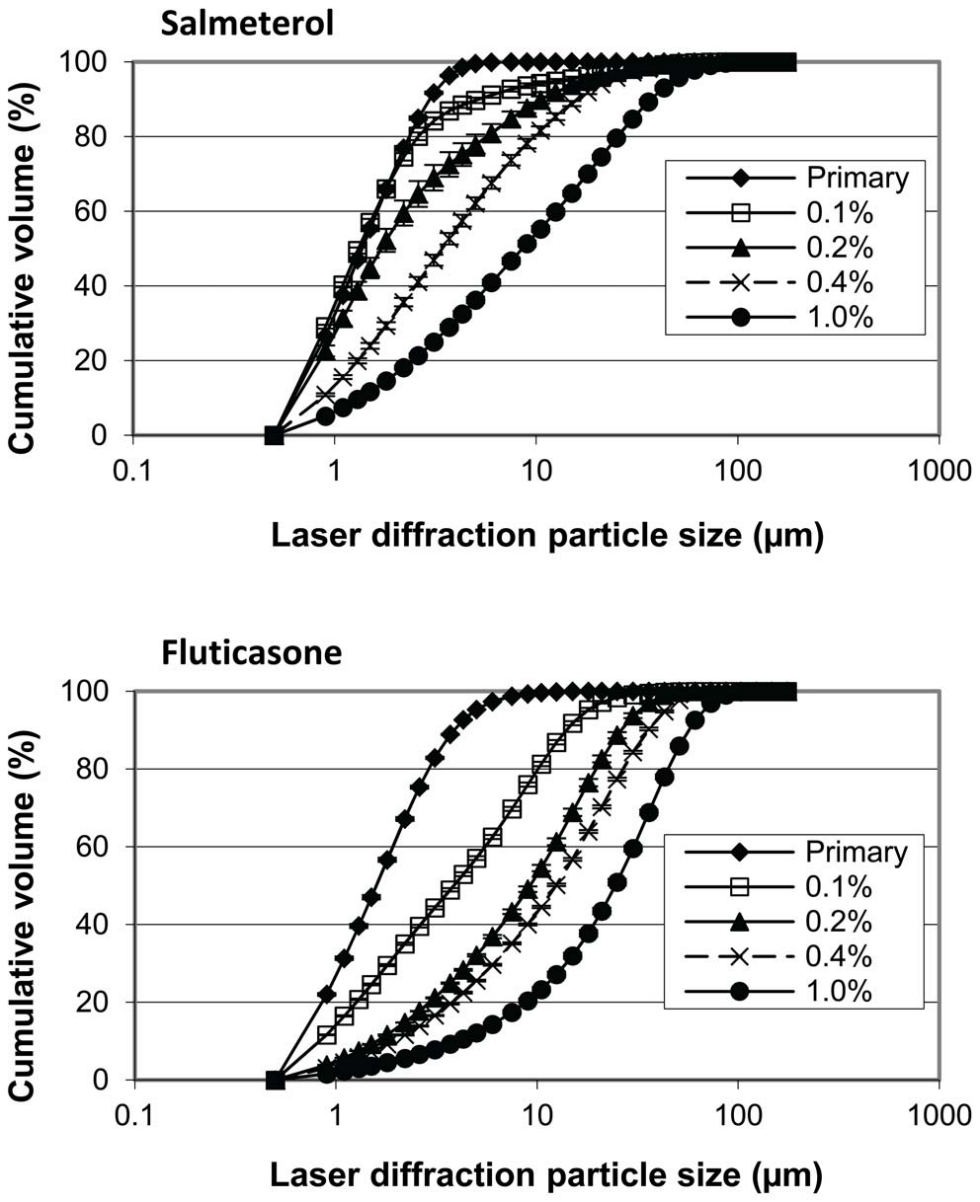
Agglomeration behaviour of salmeterol and fluticasone in the mixture with increasing drug content. Data are obtained by laser diffraction in saturated aqueous drug solutions after complete dissolution of the lactose carrier. Y-error bars represent minimum and maximum values measured (n = 2).

**Figure 5:**
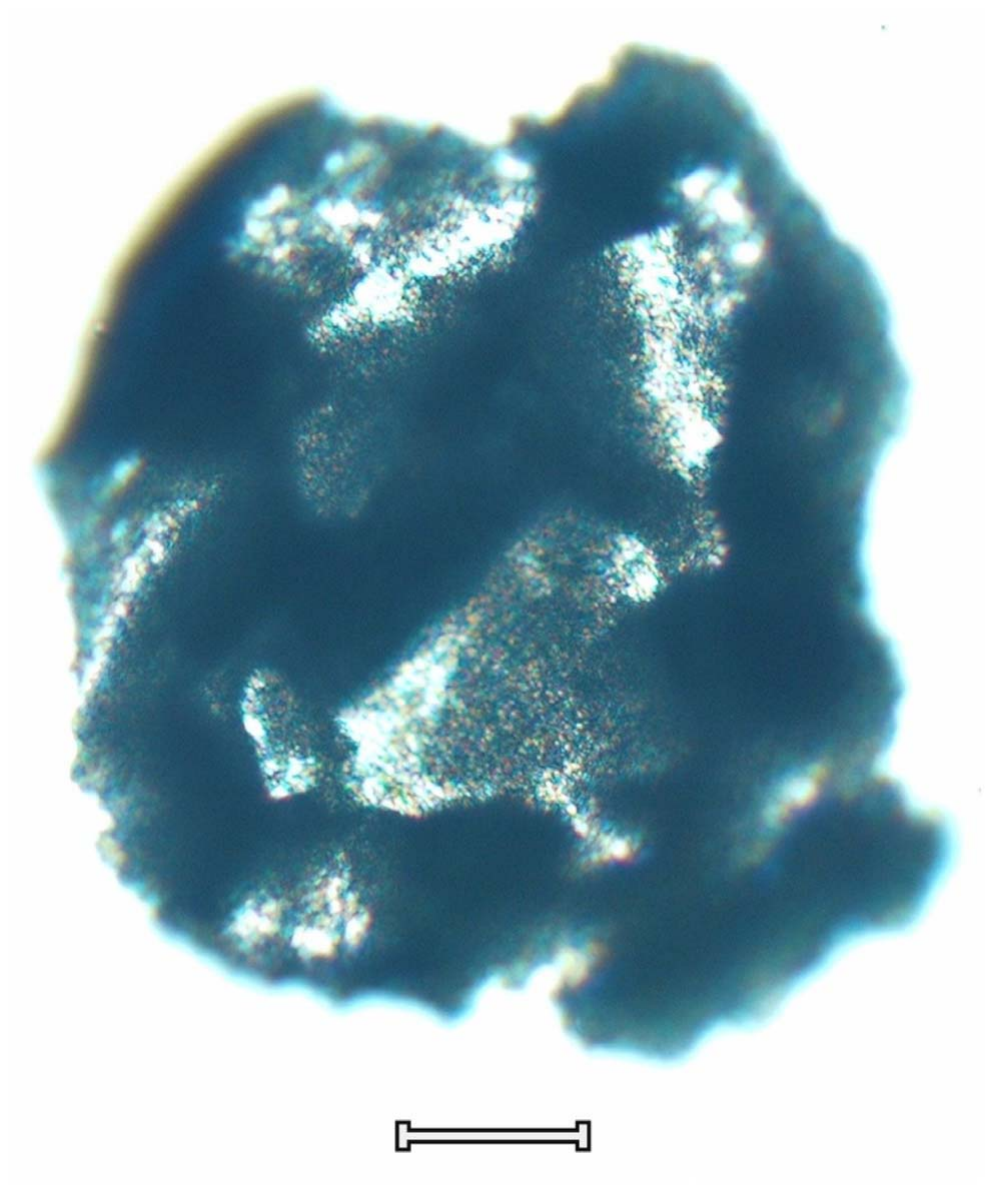
Salmeterol network in suspension after dissolution of the lactose carrier from a 4% blend. The scale bar represents 60 µm.

True density values and characteristic values for the primary PSD of the drugs used in this study are presented in Table 3. PSDs of salmeterol and fluticasone in the adhesive mixtures as released from the carrier surface during the suspension experiments are shown in Figure 4. With increasing drug content the median diameter of the drug agglomerates on the surface increases. At drug contents >1% continuous hollow drug particle networks remained in suspension after dissolution of the lactose carrier, as shown for 4% salmeterol in Figure 5. The X_50_ values of these hollow networks did not deviate much from those of the carrier crystals. Obviously, such values do not provide information on the size of detachable drug agglomerates but only on the size of the carrier particles, and therefore, these data are not shown. In many cases the shapes of the carrier particles from which the networks were detached remained recognisable after complete dissolution of the lactose.

### Drug Detachment Experiments

**Figure 6:**
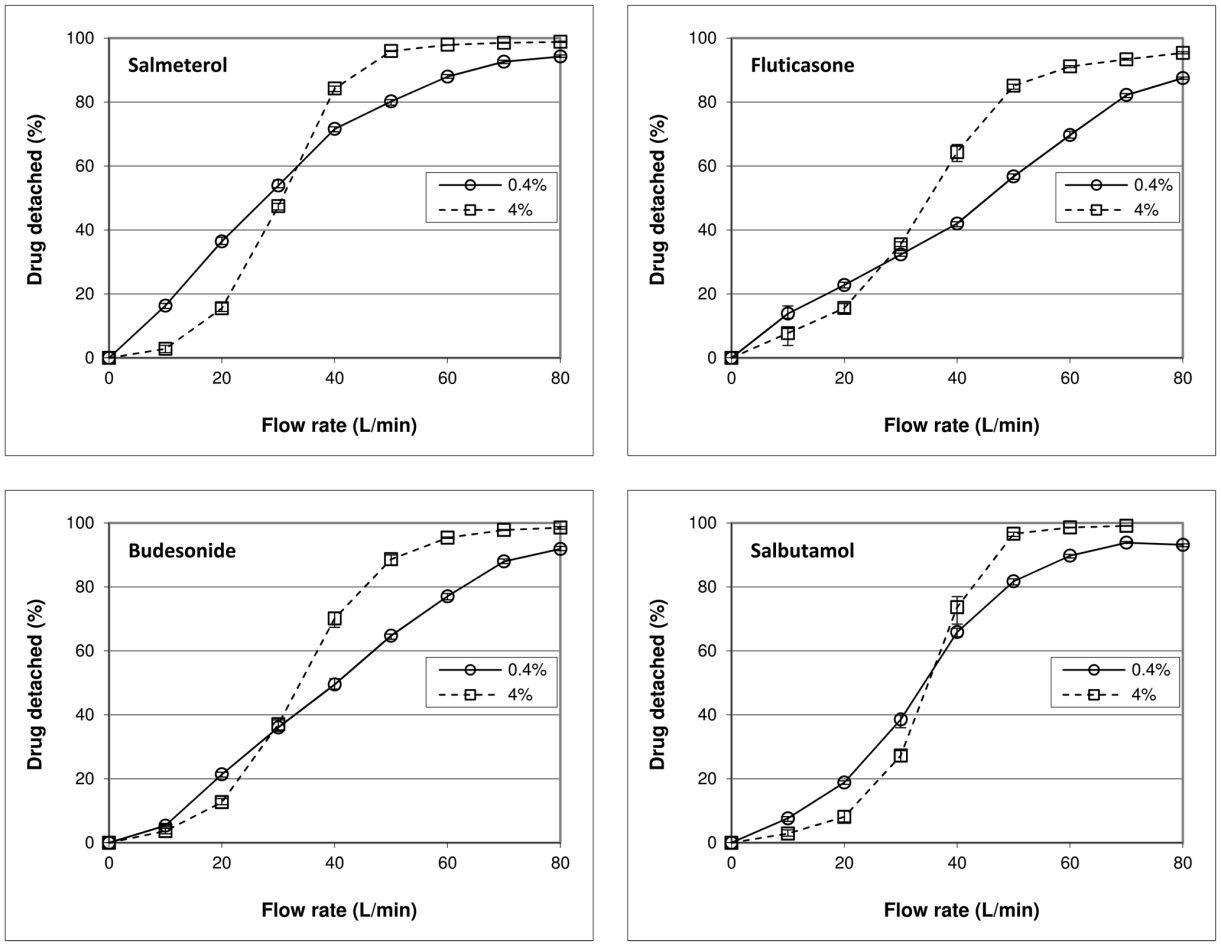
Effect of drug content on drug detachment and its dependence on flow rate and drug type. The difference between the 0.4% and 4% curves represents the effect of drug content on drug detachment at the different flow rates. The mixtures were prepared by 10 minutes of mixing. The y-error bars represent maximum and minimum values measured (n = 5).

Drug detachment as function of flow rate for the four drugs tested shows a less pronounced sigmoidal relationship at a drug content of 0.4% than for the 4% blends (Fig. 6). At flow rates below 30 to 35 L/min the relative amount of drug detached decreases when the drug content is increased from 0.4 to 4%, whereas at higher flow rates the effect is opposite. The difference in drug detachment at 20 and 50 L/min between formulations containing 0.4% and 4% of drug is statistically significant (p<0.001) for all drugs used.

**Figure 7:**
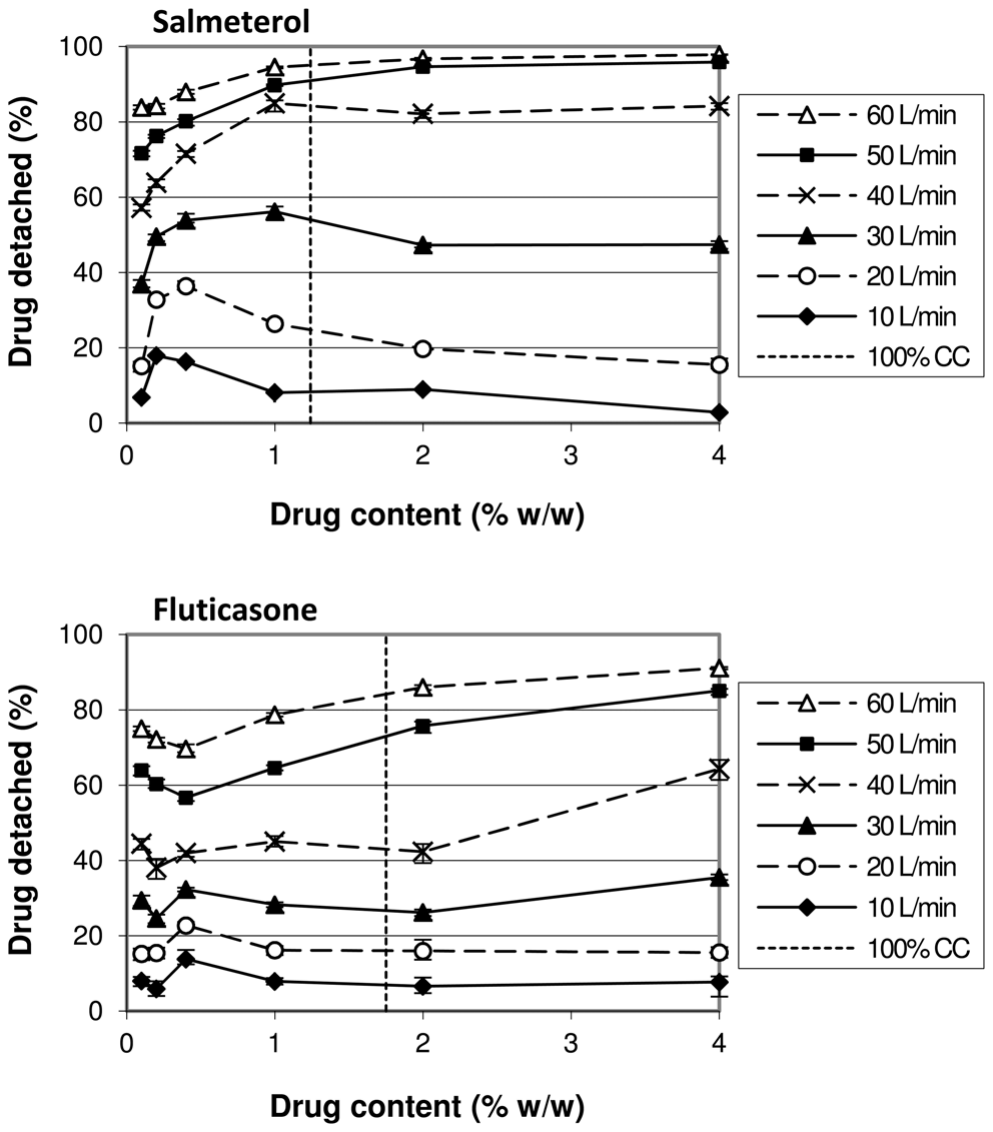
Effect of drug content on drug detachment and its dependence on flow rate and drug type. In addition to Figure 6, the dependence on the drug content range is shown. The mixtures were prepared by 10 minutes of mixing. ‘100% CC’ refers to the drug concentration corresponding to a theoretical monolayer carrier coverage. The y-error bars represent maximum and minimum values measured (n = 5).

For salmeterol and fluticasone the relationship between drug content and drug detachment at different flow rates has been examined further by preparing blends with drug contents of 0.1%, 0.2%, 1% and 2% as well. For salmeterol, an increase in drug content from 0.1% to 0.2% leads to a higher drug detachment for all flow rates up to 60 L/min (Fig. 7). At flow rates from 10 to 30 L/min a maximum drug detachment is reached between drug contents of 0.2 and 1%. Drug detachment at higher contents tends to decrease for these flow rates before reaching a constant value at the highest drug contents. At 50 and 60 L/min, drug detachment continues to increase and approaches the 100% value at a drug content of 4%. Different trends are observed for fluticasone (Fig. 7). At all flow rates drug detachment decreases initially when the drug content is increased, starting at 0.1%. After reaching a minimum value at drug contents of 0.2% (at low flow rates) and 0.4% (at high flow rates), an increase in detachment is obtained when the drug content is further increased to 0.4% (low flow rates) and 1 to 2% (high flow rates), respectively. Further increasing the drug content results in a decrease in detachment again at low flow rates, whereas detachment continues to increase marginally at 50 and 60 L/min towards a maximum value obtained at 4% drug content. It is difficult to draw unequivocal conclusions from these differences in fluticasone detachment behaviour between drug content ranges at the different flow rates, because the changes are small and stay within a narrow range of at most 8% of the initial drug content. From our experience with these drug detachment experiments we know that environmental conditions (especially relative air humidity) can very well account for differences of up to 5%, and therefore, some of these changes might not be physically relevant in relation to the changes in drug content, even if statistical significance could be proven.

**Figure 8:**
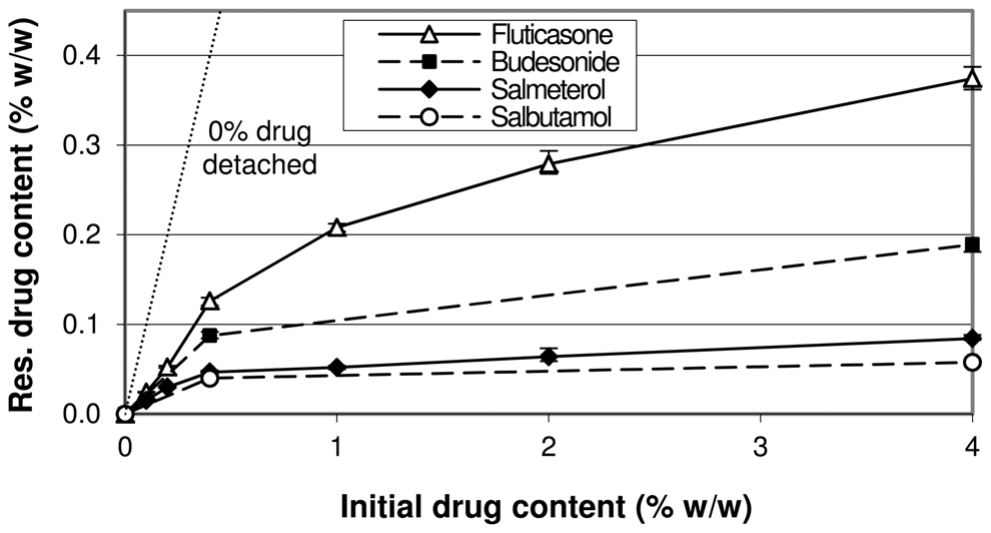
Residual drug content after a drug detachment experiment at 60 L/min as function of initial drug content. Data are a different representation of the data presented in Figures 6 and 7. The y-error bars represent minimum and maximum values measured (n = 5).

A different representation of the drug detachment data at 60 L/min from Figures 6 and 7 is given in Figure 8. With increasing drug content the slope of the relationships decreases, which means that the fraction of drug that is not detached from the carrier decreases. This may be indicative for the saturation of active sites, as explained previously for similar relationships [11]. Different plateau values for the different drugs are reached upon extrapolation in the order of fluticasone>budesonide>salmeterol>salbutamol.

**Figure 9:**
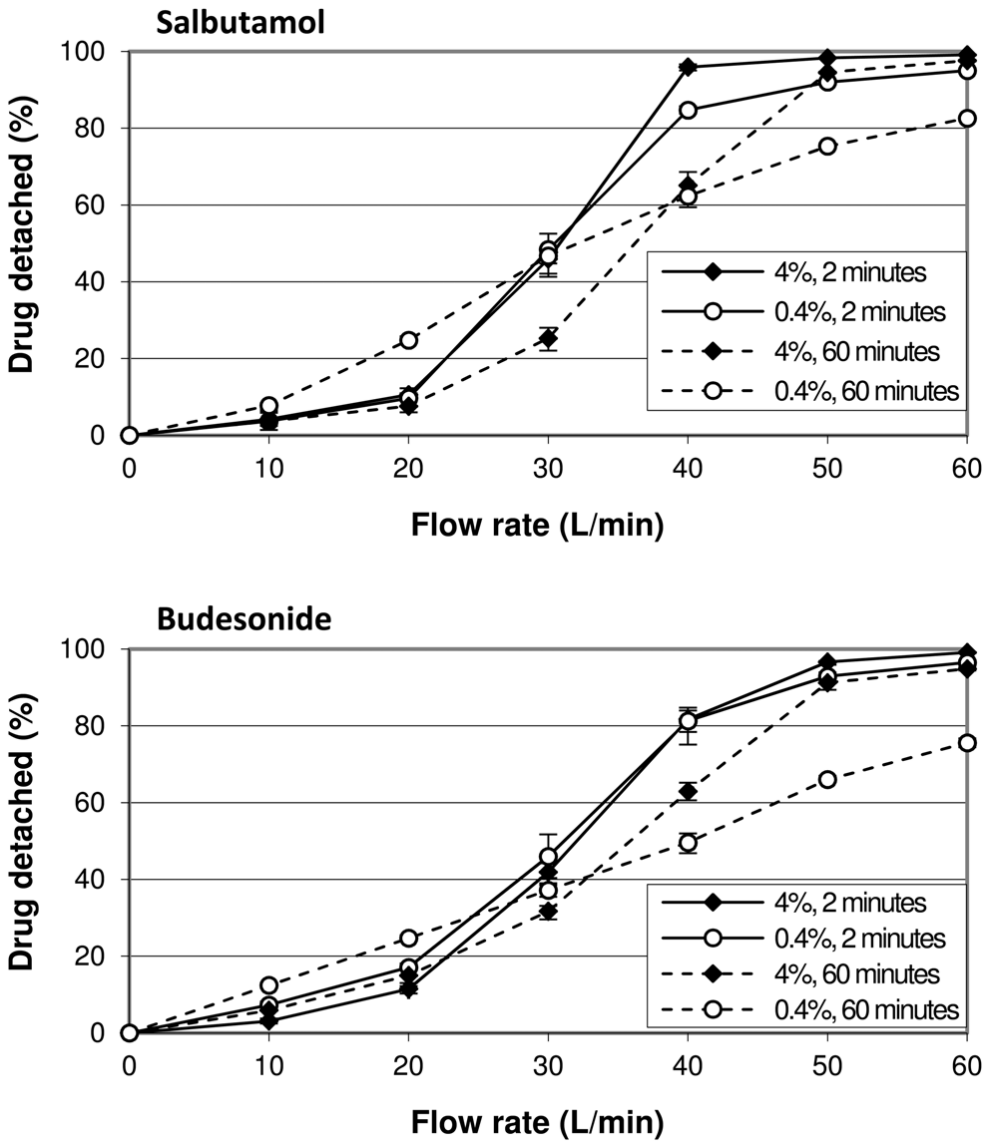
The effect of drug content on drug detachment and its dependence on mixing time. Data are shown for salbutamol and budesonide. The difference between the 0.4% and 4% curves for equal mixing times represents the effect of drug content on drug detachment at the different flow rates. The y-error bars represent maximum and minimum values measured (n = 5).

Figure 9 illustrates the role of mixing time as an interacting variable for the effect of drug content on drug detachment. After a relatively short mixing time of 2 minutes the absolute difference in drug detachment between a drug content of 0.4 and 4% is at most 11% for salbutamol and 5% for budesonide. These differences increase to 22% and 25%, respectively, after 60 minutes of mixing. At a drug content of 0.4% longer mixing results in higher drug detachment at 10 and 20 L/min for both drugs, whereas at higher flow rates drug detachment decreases. With a drug content of 4%, increasing the mixing time from 2–60 minutes results in a negative effect on drug detachment at intermediate flow rates of 30 and 40 L/min, whereas at flow rates ≤20 and ≥50 L/min the effect is negligible for both drugs.

## Discussion

### About the Occurrence of the Different Mechanisms

#### Drug agglomeration

It is reasonable to expect that more drug agglomerates (with or without lactose fines) are formed, and that their size as they are detached from the carrier during dispersion increases when the drug content in the mixture is increased. Such an expectation is supported by the data presented in Figures 2–4. This results in a change of the ratio of separation to binding forces in such a way that drug detachment is enhanced, especially for devices relying primarily on inertial separation forces such as the device used in this study [17]. An absolute quantification of this effect based on the data from this study is not possible, however. During the wet laser diffraction measurements (Fig. 4) a slow decrease of the characteristic PSD descriptors (X_10_, X_50_ and X_90_) was observed after the 12 minute period in which the lactose carrier dissolved. This is likely the result of partial deagglomeration of the drug particles in the liquid and it may be expected that this process occurs during dissolution of the lactose carrier as well. The deagglomeration process may further be influenced by dissolution of fine lactose particles that have co-agglomerated with the drug particles. The size distribution of the agglomerates could thus depend on their strength in suspension, which, in turn, is likely to depend on the degree of co-agglomeration of the drug with lactose fines. The degree of deagglomeration may vary for different drugs, which limits the direct comparison between fluticasone and salmeterol in this respect. Nevertheless, the measurements proved to be highly repeatable, as may be concluded from the y-error bars in Figure 4. In addition, the observed trend is the same for both drugs investigated. Therefore, although the data may not be correct in absolute sense, they certainly are indicative of drug particle enlargement with increasing drug content. Furthermore, the formation of drug layers and agglomerates is confirmed with SEM, especially at drug contents >1% (Fig. 2 and 3).

#### Drug compression onto the carrier surface

An increased effectiveness of press-on forces is difficult to measure, but SEM images may be used to quantify at least the drug content above which the propensity for this effect will increase. The complete filling of irregularities in the carrier surface is approached within the range of drug contents between 0.4 and 1% (Fig. 2). From this concentration upward the efficacy of the mixing forces as press-on forces is likely to increase, which, as explained in the introduction, is expected to contribute in a negative way to the effect of drug content on drug detachment during inhalation.

#### Saturation of active sites

Based on the results presented in Figure 8 and the data presented in previous studies [10], [11], the occurrence of the saturation of active sites with an increasing drug content seems very plausible. However, at low drug contents (i.e. below theoretical monolayer carrier coverage) this effect is expected to be strongly dependent on the degree of preferential occupation of active sites: if no preferential occupation of active sites occurs during mixing, then saturation of active sites is not possible within this drug content range. Dispersion experiments do not provide the ability to distinguish between the saturation of active sites, agglomeration effects and press-on effects. Therefore, to definitively prove the occurrence of the saturation of active sites it is necessary to measure differences in the relative amount of drug attached to active sites (for example the non-detached drug fraction after dispersion at 60 L/min) between the different drug contents in the absence of agglomeration and press-on effects. Because in this study at least agglomeration already occurs from the lowest drug content used upward (as discussed), this is not possible. Strong (indirect) indications for the occurrence of the saturation of active sites as a result of changes in drug content have never been presented along with definitive proof of the absence of other mechanisms either. Therefore, although the saturation of active sites is very likely to occur, it cannot be proven beyond reasonable doubt with the current data presented and the relevance of this mechanism to the overall effect of drug content in this study remains uncertain.

**Figure 10:**
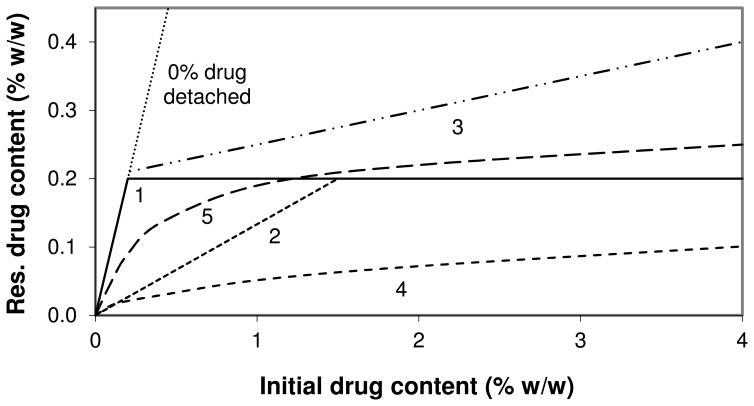
Relationships between residual and initial drug content for different hypothetical scenarios. Residual drug content = drug content after a drug detachment experiment at 60 L/min. Drug contents that equal the maximum binding capacity of active sites and monolayer coverage are assumed to be 0.2% and 1.5%, respectively. The relationships represent: **1)** completely preferential occupation of active sites, absence of effects from agglomeration, press-on forces and layer formation; **2)** same as situation 1 with completely non-preferential occupation of active sites; **3)** same as situation 1 with effects from press-on forces and layer formation; **4)** same as situation 1 with a high degree of agglomeration; **5)** intermediate situation with unknown balance of mechanisms.

To further illustrate this problem, several hypothetical relationships between the residual drug content on the carrier after a drug detachment experiment at 60 L/min and the initial drug content in the blend are displayed in Figure 10. For the purpose of this example we define active sites as sites where drug particles can only be detached at flow rates >60 L/min through the inhaler used in this study. Furthermore, we assume that 100% monolayer coverage of the carrier corresponds to a drug content of 1.5% and that the maximum binding capacity of active sites for the primary drug particles corresponds to a drug content of 0.2%. In situation 1 the preferential occupation of active sites is complete, no agglomeration or press-on effects occur and multilayer coverage of the carrier by the drug does not result in the formation of new active ‘apparent carrier surface sites’ by underlying drug layers. Under such conditions the saturation of active sites is characterised by a relationship that follows the ‘0% drug detached’ line (for which y = x) until a plateau value is reached that is equal to the binding capacity of active sites. The saturation of active sites results in a positive effect on drug detachment at drug contents >0.2%. In situation 2 completely non-preferential occupation of active sites occurs (i.e. a random distribution of drug particles over all carrier sites is obtained), which causes active sites to be saturated only when the complete carrier surface is covered with a drug monolayer. In this situation the saturation of active sites results in a positive effect on drug detachment only at initial drug contents >1.5% (i.e. the concentration at which the complete surface is covered). In situation 3 completely preferential occupation of active sites in combination with an increasing effectiveness of press-on forces with increasing drug content causes an increasing drug mass to be attached to ‘pseudo-active sites’ [19], thereby gradually increasing the total binding capacity of active sites. In the case of multilayer coverage of the carrier, underlying drug layers may also form new active sites to increase their total binding capacity. This results in a higher drug concentration after detachment at drug contents >0.2% compared to relationship 1. Relationship 4 represents the situation in which drug agglomeration dominates the overall effect of drug content: a higher agglomeration potential of the drugs with increasing drug content causes the ratio of separation to binding force to increase due to agglomeration for an increasing fraction of the drug mass. As a result, the saturation content of active sites (for the primary drug particles) may never be reached after detachment and saturation of active sites does not contribute to improved drug detachment with increasing drug content. Relationship 5 displays an intermediate situation between situations 3 and 4. The lower slope with increasing drug content is representative for a larger fraction of the drug mass being detached. In the absence of the exact binding capacity of active sites as a reference (which is the situation in practice), situation 5 cannot be distinguished from situation 4 when regarded individually. This means that the increased drug detachment may be completely the result of either agglomeration effects, or the saturation of active sites (with a gradually decreasing degree of preferential occupation of active sites with increasing drug content) or a combination of both. Therefore, only in the absence of agglomeration effects such a relationship, which is representative of those experimentally determined for the different drugs (Fig. 8), would be proof of the occurrence of the saturation of active sites.

### About the Variables that Interact with the Effect of Drug Content

#### The flow rate

The results presented in Figure 6 show that flow rate interacts with the effect of drug content in a quantitative and qualitative way. This could be explained by the flow rate causing a shift in the balance of the different mechanisms behind the effect of drug content on drug detachment. At low flow rates (<30–35 L/min) the negative effect from press-on effects has to dominate any positive contribution from agglomeration effects or the saturation of active sites. At flow rates above 30–35 L/min a dominance of agglomeration effects and the saturation of active sites over press-on effects gives an opposite result. In the following section it will be discussed that this finding is strongly dependent on the chosen drug content range of 0.4 to 4%.

Because flow rate does not influence the properties of the powder mixtures, the shift in the balance of the different mechanisms concerns a change in their significance to drug detachment. For example, a decrease in the drug fraction that is only detached at flow rates >60 L/min due to the saturation of active sites does not necessarily increase the fraction that is detached at flow rates <30 L/min. It may also just increase the fraction that is exclusively detached at flow rates between 30 and 60 L/min. In that case, the saturation of active sites would be significant to drug detachment only at flow rates >30 L/min. In a following article (in preparation) the interacting effect of flow rate will be discussed and explained in more detail.

#### The drug content range

As explained, the occurrence of press-on effects and the saturation of active sites may be strongly dependent on the range over which the drug content is changed. It therefore determines their balance and thus the overall effect of drug content on drug detachment. Strictly the drug content range is not an interacting variable for the effect of drug content, but a specific property of the variable itself. Therefore, this interaction is an example of a conditional effect [20]. Its occurrence is confirmed by the results presented in Figure 7, which shows both quantitative and qualitative interactions. The difference between salmeterol and fluticasone in this respect shows also a drug specific influence.

In the discussion about the occurrence of the three mechanisms it was reasoned that agglomeration is likely to occur starting at the lowest contents used, and that press-on effects become relevant from drug contents of 0.4% upwards. The concentration dependent salmeterol detachment at flow rates up to and including 30 L/min presented in Figure 7 can therefore be explained with an initial dominance of agglomeration effects (causing the initial increase in drug detachment between 0.1 and 0.2–0.4%) followed by a dominance of press-on effects with increasing drug content (causing the decrease in drug detachment). Apparently, at higher flow rates separation energies have become so high that the increase in binding energy by press-on effects has become insignificant. As discussed, the saturation of active sites may contribute to the overall effect too, but their relevance is uncertain.

The trends observed for fluticasone detachment within the same drug content range are not the same (Fig. 7). Considering the data presented in Figure 4, agglomerate formation also occurs for fluticasone. Apparently, the effect from agglomeration is balanced by the effect of press-on forces and the saturation of active sites in a different way as for salmeterol. A more effective redistribution of fluticasone towards active sites, a different interaction with the lactose fines that are still present on the carrier surface after sieving or a higher effectiveness of equally high press-on forces due to drug specific characteristics (which will be discussed further on) may explain this difference.

#### The mixing time

The data presented in Figure 9 show that mixing time interacts with the effect of drug content on drug detachment in a quantitative way. The small difference in drug detachment between 0.4% and 4% after 2 minutes of mixing for both drugs indicates that mixing is the driving force for the three mechanisms discussed. Within such a short mixing time insufficient ‘chance effects’ (i.e. drug redistribution towards active sites, drug de- or re-agglomeration and compression of the drug particles onto the carrier surface) seem to have occurred to significantly influence drug detachment. Alternatively, if these chance effects would have been sufficient to induce significant agglomeration and press-on effects and the saturation of active sites between 0.4 and 4% after 2 minutes of mixing, they would have to counteract each other over the range of flow rates that is applied. This is not very likely. Therefore, the mechanisms seem to become more significant during prolonged mixing but to a different extent, which magnifies any overall effect that results from their balance. It should be realised that the data from Figures 6 and 7 (obtained with 10 minutes of mixing) are therefore also to a great extent the result of the chosen mixing time, at least with respect to the magnitude of the observed effects.

An increase in drug content from 0.4–4% causes a notable interaction with the effect of mixing time on drug detachment too. At higher concentrations, higher flow rates are required to achieve the same relative amount of drug detached when mixing is prolonged. This negative effect is likely the result of a dominance of press-on effects. For the lower drug content, the effect of prolonged mixing more explicitly depends on the flow rate. The negative effect at the higher flow rates can be explained by a dominance of press-on effects and the redistribution of drug particles towards active sites. The positive effect at low flow rates seems contradictory to the redistribution of drug particles towards active sites, but it can be explained by agglomeration effects and the migration of drug particles towards sites of low binding activity nonetheless. The mixing forces cause partial agglomeration of the drug particles with each other and with lactose fines. These resulting larger particles are most easily detached due to a larger ratio of separation to binding force. Hence, they are likely first detached at low flow rates. Drug particles also find shelter from collisional and frictional mixing forces in carrier surface irregularities without necessarily being attached with a high binding force. Because the drug particles do not find shelter from inertial separation forces at these sites during inhalation, migration of particles towards these sites may result in a higher ratio of separation to binding force too and can thus be regarded as the redistribution of drug particles towards sites with a low activity. This process can very well occur concurrently with the redistribution of drug particles towards highly active sites. Which mechanism is most relevant for the effect of mixing time on drug detachment for mixtures that contain low drug contents will be the subject of a future study.

#### The type of drug

Previously presented values for the cohesion-adhesion balance (CAB) of salbutamol, salmeterol, fluticasone and budesonide in combination with alpha lactose monohydrate are 0.63, 2.39, 0.22 and 0.82 respectively [21], [22]. Although it has been described that CAB-values can be highly batch dependent [23], it is expected that the choice for the different drugs brings about a difference in adhesion and cohesion characteristics. This is supported by Figure 8, in which a higher plateau value indicates that the average binding force for that drug to lactose is higher, or that the average separation force is lower due to a lower tendency to form agglomerates (or both). In this respect the low CAB-value (0.22) of fluticasone could be indicative of a high intrinsic adhesion to lactose, whereas salmeterol (2.39) would be more prone to form agglomerates which are more easily detached [24]. However, the literature CAB-values for budesonide and salbutamol are not in agreement with the trends observed in Figure 8, and neither are the observations with SEM regarding the degree of agglomeration in Figure 3 (visually there seems to be hardly a difference in the degree of agglomeration between the different drugs). The different plateau values for the drugs could also be the result of a difference in their average particle mass, which is the result of the PSD of the drug and its density. In a classifier based test inhaler the ratio of separation to binding force is lower for lighter particles and they will therefore be less likely to detach during inhalation. This would result in a higher plateau value in Figure 8. However, the drug with the highest calculated average particle mass (i.e. fluticasone, Table 3) also has the highest plateau value, and therefore, differences in the average particle mass neither explain the different plateau levels. Other drug specific characteristics that are of influence on the adhesive and cohesive interactions, such as the observed differences in particle shape and roughness, but also surface energy, moisture adsorption, deformability (e.g. Young’s modulus, Poisson ratio) and electrostatic properties are thus more likely to be the cause of the observed differences.

Regardless of the underlying mechanism, the difference in cohesion and adhesion characteristics between the drugs is expected to cause a different balance of mechanisms with a change in drug content. For example, the saturation of active sites may be more pronounced for strongly adhesive drugs, especially when the drug content is increased beyond monolayer coverage of the carrier surface. For strongly cohesive drugs, however, agglomeration effects may be more pronounced. The data presented in Figures 6 and 9 show that the drug dependence of the effect of a change in drug content is expressed not so much in the direction as in the magnitude of the effect. However, from the data presented in Figure 7 it follows that an independence of the direction of the effect from the drug properties can be a result of the chosen drug content range. For example, a change in drug content from 0.1–0.4% results in opposite effects on drug detachment between salmeterol and fluticasone at flow rates of 40 L/min and higher. Therefore, the type of drug causes both qualitative and quantitative interactions with the effect of drug content, depending on the drug content range.

#### Other variables

The results from this study may be a consequence of the choice for other variables as well. For example, the dispersion principle of the test inhaler used relies mostly on inertial separation forces. This likely enhances the contribution of agglomeration effects to the overall balance of effects. Furthermore, the coarse crystalline carrier fraction used may cause higher press-on forces than a finer or granulated carrier fraction. The negative contribution of an increased effectiveness of these forces to the overall balance of mechanisms in this study may therefore have been more dominant than will be the case for other carrier materials. The opposite may be true for the low shear mixing process and small batch size that was used. Finally, the drug contents at which certain effects have been observed are likely biased by the presence of lactose fines on the carrier surface after the sieving procedure. This may especially be true at low drug contents, where the lactose fines comprise a significant part of the total fine particle mass.

### Practical Implications

Based on the results from this study it is conceivable that the differences in drug content between commercially available formulations result in a different dispersion behaviour. This is especially relevant for drugs from the same manufacturer that are available in different dose strengths. Furthermore, a drug to lactose ratio of 1∶67.5 (1.46%) is not used in any of the formulations presented in Tables 1 and 2, which should stimulate researchers to use other than historical reasons for the choice of a certain drug to lactose ratio. Examples could be a certain desired carrier coverage (monolayer or multilayer), the desire to make use of the sheltering capacity of carrier surface irregularities or the approximate maximum binding capacity of active sites for the carrier-drug combination that is being used. In addition, it follows from the discussion that the drug content can greatly affect the balance of different mechanisms. This has to be taken into account when investigating the influence of other variables on the dispersion performance of adhesive mixtures.

In explaining the effect of added lactose fines on the dispersion performance of adhesive mixtures, the same mechanisms as for the effect of drug content may be expected. This means that also quantitative and qualitative interactions of flow rate with the effect of added fines can occur. This was demonstrated previously for lactose fines with a similar size distribution to the drug particles [20].

### Conclusions

The effects of drug content on drug particle detachment from lactose carriers can be explained with a balance between agglomeration effects, press-on effects and the saturation of active sites. The flow rate, the type of drug, mixing time and the drug content range are shown to cause quantitative and qualitative interactions, likely by shifting the balance between the mechanisms in play. Our findings explain conflicting results that were previously reported in literature regarding the effect of drug content on dispersion performance. They furthermore irrefutably show that the choice for variables other than the ones under investigation should not be based on historical reasons, but rather on how they may interact with the primary variable(s) of interest. A higher drug content may lower the significance of a mechanism such as the saturation of active sites and can therefore shift the balance of effects of other variables towards agglomeration or press-on effects, as is shown for the effect of mixing time in this study.

## References

[1] Timsina MP, Martin GP, Marriott C, Ganderton D, Yianneskis M (1994) Drug delivery to the respiratory tract using dry powder inhalers. International Journal of Pharmaceutics 101: 1–13.

[2] Staniforth JN (1981) Total Mixing. Int J Pharm Tech & Prod Mfr 2: 7–12.

[3] Staniforth JN (1987) British Pharmaceutical Conference Science Award Lecture 1986: Order out of chaos. Journal of Pharmacy and Pharmacology 39: 329–334. 2886579 10.1111/j.2042-7158.1987.tb03393.x

[4] Hersey JA (1975) Ordered mixing: A new concept in powder mixing practice. Powder Technology 11: 41–44.

[5] Bryan L, Rungvejhavuttivittaya Y, Stewart PJ (1979) Mixing and demixing of microdose quantities of sodium-salicylate in a direct compression vehicle. Powder Technology 22: 147–151.

[6] Kulvanich P, Stewart PJ (1987) The effect of particle size and concentration on the adhesive characteristics of a model drug-carrier interactive system. Journal of Pharmacy and Pharmacology 39: 673–678. 2890731 10.1111/j.2042-7158.1987.tb06968.x

[7] Steckel H, Markefka P, teWierik H, Kammelar R (2004) Functionality testing of inhalation grade lactose. European Journal of Pharmaceutics and Biopharmaceutics 57: 495–505. 15093599 10.1016/j.ejpb.2003.12.003

[8] Steckel H, Muller BW (1997) In vitro evaluation of dry powder inhalers.2. Influence of carrier particle size and concentration on in vitro deposition. International Journal of Pharmaceutics 154: 31–37.

[9] Le VNP, Hoang Thi TH, Robins E, Flament MP (2012) In vitro evaluation of powders for inhalation: The effect of drug concentration on particle detachment. International Journal of Pharmaceutics 424: 44–49. 22207163 10.1016/j.ijpharm.2011.12.020

[10] Young PM, Edge S, Traini D, Jones MD, Price R, et al (2005) The influence of dose on the performance of dry powder inhalation systems. International Journal of Pharmaceutics 296: 26–33. 15885452 10.1016/j.ijpharm.2005.02.004

[11] de Boer AH, Dickhoff BHJ, Hagedoorn P, Gjaltema D, Goede J, et al (2005) A critical evaluation of the relevant parameters for drug redispersion from adhesive mixtures during inhalation. International Journal of Pharmaceutics 294: 173–184. 15814242 10.1016/j.ijpharm.2005.01.035

[12] Louey MD, Stewart PJ (2002) Particle Interactions Involved in Aerosol Dispersion of Ternary Interactive Mixtures. Pharmaceutical Research 19: 1524–1531. 12425471 10.1023/a:1020464801786

[13] de Boer AH, Hagedoorn P, Gjaltema D, Lambregts D, Irngartinger M, et al (2004) The Mode of Drug Particle Detachment from Carrier Crystals in an Air Classifier-Based Inhaler. Pharmaceutical Research 21: 2167–2174. 15648247 10.1007/s11095-004-5171-6

[14] de Boer AH, Hagedoorn P, Gjaltema D, Lambregts D, Irngartinger M, et al (2004) The Rate of Drug Particle Detachment from Carrier Crystals in an Air Classifier-Based Inhaler. Pharmaceutical Research 21: 2158–2166. 15648246 10.1007/s11095-004-7668-4

[15] Dickhoff BHJ, de Boer AH, Lambregts D, Frijlink HW (2003) The effect of carrier surface and bulk properties on drug particle detachment from crystalline lactose carrier particles during inhalation, as function of carrier payload and mixing time. European Journal of Pharmaceutics and Biopharmaceutics 56: 291–302. 12957644 10.1016/s0939-6411(03)00109-7

[16] Lam KK, Newton JM (1991) Investigation of applied compression on the adhesion of powders to a substrate surface. Powder Technology 65: 167–175.

[17] de Boer AH, Hagedoorn P, Gjaltema D, Goede J, Frijlink HW (2003) Air classifier technology (ACT) in dry powder inhalation: Part 1. Introduction of a novel force distribution concept (FDC) explaining the performance of a basic air classifier on adhesive mixtures. International Journal of Pharmaceutics 260: 187–200. 12842339 10.1016/s0378-5173(03)00250-3

[18] De Koning JP (2001) Dry powder inhalation. The Netherlands: University of Groningen.

[19] Dickhoff BHJ (2006) Adhesive mixtures for powder inhalation. The Netherlands: University of Groningen.

[20] de Boer AH, Chan HK, Price R (2012) A critical view on lactose-based drug formulation and device studies for dry powder inhalation: Which are relevant and what interactions to expect? Advanced Drug Delivery Reviews 64: 257–274. 21565232 10.1016/j.addr.2011.04.004

[21] Jones M, Hooton J, Dawson M, Ferrie A, Price R (2008) An Investigation into the Dispersion Mechanisms of Ternary Dry Powder Inhaler Formulations by the Quantification of Interparticulate Forces. Pharmaceutical Research 25: 337–348. 17952568 10.1007/s11095-007-9467-1

[22] Hooton JC, Jones MD, Price R (2006) Predicting the behavior of novel sugar carriers for dry powder inhaler formulations via the use of a cohesive–adhesive force balance approach. Journal of Pharmaceutical Sciences 95: 1288–1297. 16637052 10.1002/jps.20618

[23] Price R (2010) Low and High Energy Blending. Presentation at conference: Lactose as a carrier for inhalation products. Parma.

[24] Jones MD, Harris H, Hooton JC, Shur J, King GS, et al (2008) An investigation into the relationship between carrier-based dry powder inhalation performance and formulation cohesive-adhesive force balances. European Journal of Pharmaceutics and Biopharmaceutics 69: 496–507. 18191553 10.1016/j.ejpb.2007.11.019

